# Revascularization of the Superficialized Brachial Artery

**DOI:** 10.3400/avd.oa.25-00093

**Published:** 2025-12-17

**Authors:** Shunichiro Fujioka, Kenjiro Sakaki

**Affiliations:** 1 Department of Vascular Surgery, Tachibanadai Hospital, Yokohama, Kanagawa, Japan; 2 Department of Cardiovascular Surgery, Shonan Atsugi Hospital, Atsugi, Kanagawa, Japan

**Keywords:** superficialized brachial artery, aneurysm, revascularization, hemodialysis access, autologous vein bypass

## Abstract

**Objectives:**

The superficialized brachial artery (SBA) is an important alternative vascular access for hemodialysis patients when autogenous vein fistula creation is not feasible. However, repeated puncture of the SBA can lead to severe complications such as aneurysm formation, pseudoaneurysm, and infection. This study aimed to review surgical strategies for revascularization and infection control in SBA aneurysms.

**Methods:**

We retrospectively analyzed 8 cases of SBA aneurysms treated at our institution between November 2020 and June 2025. Patient demographics, comorbidities, surgical procedures, and outcomes were evaluated based on medical records and follow-up data.

**Results:**

Patients ranged in age from 43 to 81 years and had been on dialysis for an average of 19 years. Six aneurysms were ruptured, and 5 were associated with infection. Brachial artery bypass was the most common procedure, performed in 6 patients using autologous veins or prosthetic grafts. One patient underwent fistula closure with a bovine pericardial patch, and another received direct arterial anastomosis. In infected cases, autologous vein bypass or aneurysm resection with direct anastomosis was performed after thorough debridement. All patients maintained adequate dialysis access postoperatively.

**Conclusions:**

Revascularization of the SBA using autologous vein bypass is effective for managing aneurysms, especially in infected cases. Careful infection control and individualized surgical planning are essential for maintaining safe dialysis access and preserving limb function.

## Introduction

The superficialized brachial artery (SBA) is a peripheral arterial blood access technique of choice for patients who cannot undergo autogenous vein shunt creation or who have a history of heart failure. While the SBA enables high flow rates through direct arterial puncture, repeated punctures pose significant risks, including aneurysm formation, pseudoaneurysm, perforation, and infection. Superficial lesions are especially vulnerable, and when infection or pseudoaneurysm occurs at the puncture site, revascularization following lesion resection is required to preserve the affected limb.

We performed resection and revascularization for SBA aneurysms in 8 patients and herein report our findings and discuss treatment strategies for SBA-related complications.

## Materials and Methods

A retrospective case series was conducted on 8 SBA aneurysms treated between November 2020 and June 2025. Medical records and postoperative follow-up data were analyzed.

## Results

Patients ranged in age from 43 to 81 years (mean 62 years) and included 5 males and 3 females (**Table 1**). Dialysis duration ranged from 8 to 32 years (mean 19 years). The mean operative time was 166 ± 54 minutes. Preoperative comorbidities included hypertension (n = 6), diabetes (n = 2), prior myocardial infarction (n = 2), prior stroke (n = 2), arteriosclerosis obliterans (n = 2), systemic lupus erythematosus (SLE) (n = 2), and atrioventricular block requiring pacemaker implantation (n = 1).

**Table 1:** Patient characteristics, comorbidities, surgical procedures, and materials used

Case	Age	Gender	Dialysis (years)	HTN	DM	HL	OMI	Stroke	PAD	Other	Rupture	Infection	Procedure	Material
1	68	M	16	+	−	−	−	−	−	−	−	−	Bypass	Prosthetic graft
2	59	F	23	+	−	−	−	−	−	SLE	+	+	Fistula closure	Bovine pericardium
3	59	M	13	+	−	+	−	+	−	−	+	+	Bypass	Cephalic vein
4	51	M	8	+	+	−	+	−	+	−	−	−	Bypass	Prosthetic graft
5	59	F	23	+	−	−	−	−	−	SLE	+	+	Bypass	Great saphenous vein
6	43	F	12	−	+	−	−	+	−	−	+	+	Bypass	Great saphenous vein
7	81	M	32	+	−	−	−	−	−	−	+	+	Direct anastomosis	—
8	77	M	23	−	−	−	+	−	+	PM	−	−	Bypass	Prosthetic graft

HTN: hypertension; DM: diabetes mellitus; HL: hyperlipidemia; OMI: old myocardial infarction; PAD: peripheral artery disease; SLE: systemic lupus erythematosus; PM: pacemaker implantation; M: male; F: female

Six lesions were ruptured brachial artery aneurysms, and 5 were associated with infection.

Brachial artery bypass was the most common procedure, performed in 6 cases. Conduits included the great saphenous vein (n = 2), radial skin vein of the upper arm (n = 1), and prosthetic grafts (n = 3). In addition, 1 fistula closure using bovine pericardium and 1 aneurysmectomy with direct arterial anastomosis were performed.

When infection was present or when the distal bypass extended beyond the elbow, an autologous vein graft was used. One pseudoaneurysm with a fistula was closed with a bovine pericardial patch.

To ensure continued dialysis access, new autogenous vein shunts were created on the contralateral arm in 3 cases and on the ipsilateral arm in 1 case, while prosthetic graft shunts were created on the contralateral arm in 1 case and on the ipsilateral arm in 2 cases. In 1 case, the brachial artery was re-superficialized distal to the wound after fistula closure.

Five cases involved infection, mainly due to bacterial contamination at the puncture site or pseudoaneurysm formation. These cases were treated with resection of the infected segment and bypass using an autogenous vein or direct arterial anastomosis. There were no perioperative deaths, and no graft occlusion was observed during the follow-up period.

### Representative cases

#### Case 1

**Fig. 1:**
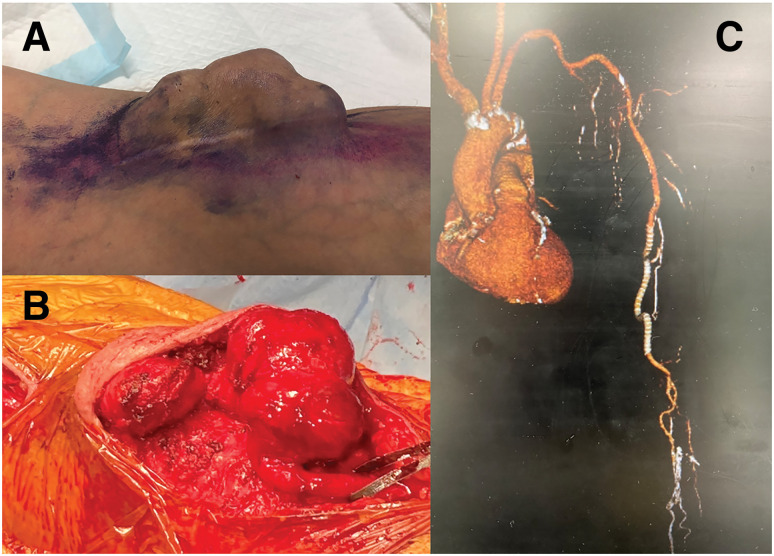
(**A**) Large, painful SBA aneurysm. (**B**) Intraoperative view showing a large mass at the puncture site. (**C**) Contrast CT after graft replacement. SBA: superficialized brachial artery; CT: computed tomography

A 68-year-old male on dialysis for 16 years with hypertension presented with an SBA aneurysm showing saccular enlargement and puncture site pain (**Fig. 1**). With no rupture or infection evident, an autogenous vein shunt was created in the contralateral arm, and the aneurysm was repaired with an artificial graft, with distal anastomosis to the brachial artery above the elbow.

#### Case 2

**Fig. 2:**
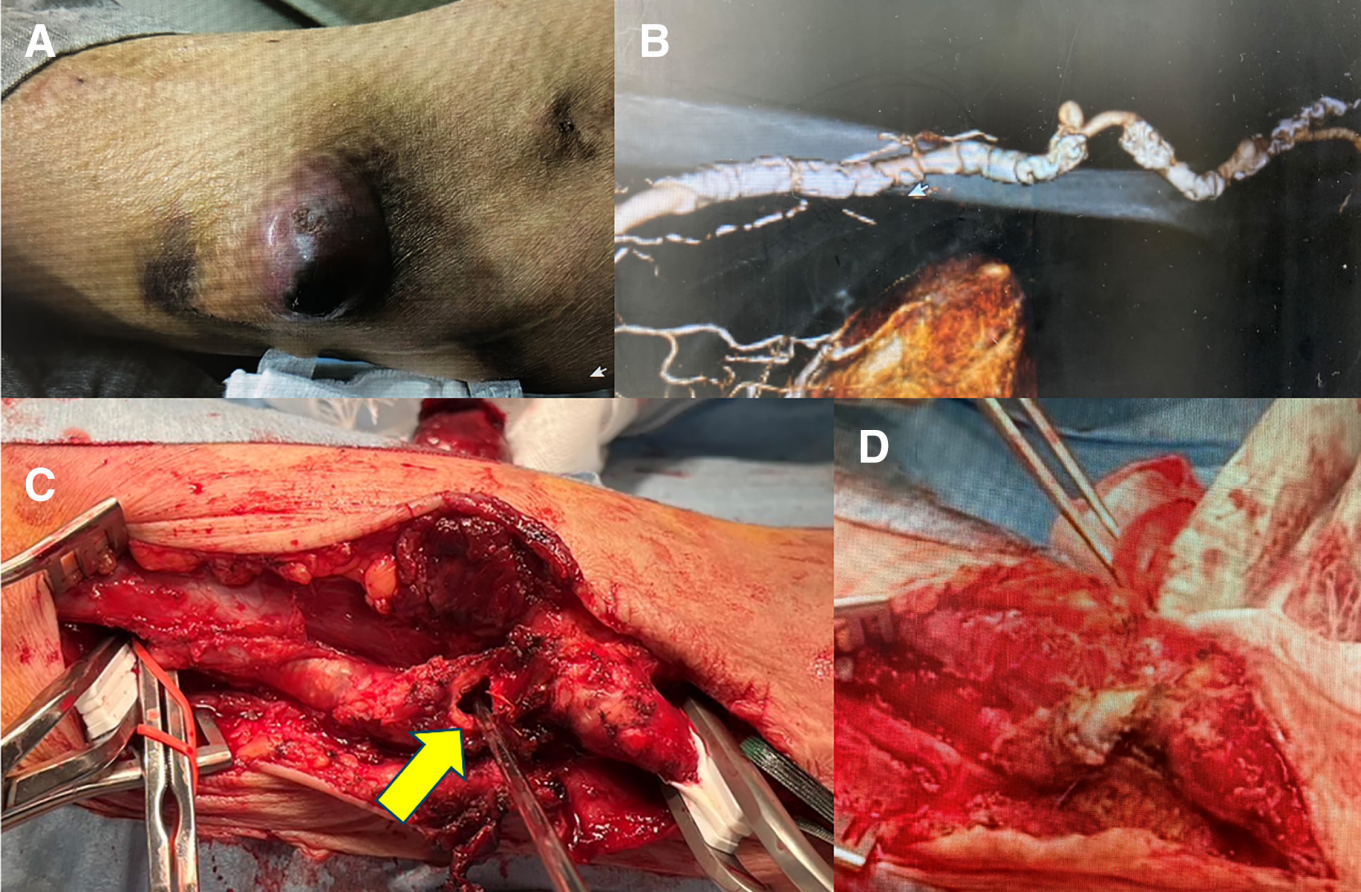
(**A**) Erythematous SBA aneurysm with pus. (**B**) CT showing severe circumferential calcification. (**C**) Large SBA fistula. (**D**) Fistula closed with bovine pericardial patch. SBA: superficialized brachial artery; CT: computed tomography

A 59-year-old female on dialysis for 23 years with SLE and hypertension presented with an SBA aneurysm with redness and purulent discharge at the puncture site (**Fig. 2**). She was diagnosed with a ruptured, infected SBA aneurysm and underwent emergency surgery. The calcified artery was closed with a bovine pericardial patch. Early postoperative recovery was good; however, due to the superficial position of the artery, a cutaneous fistula developed at the patch site 4 months later.

#### Case 3

**Fig. 3:**
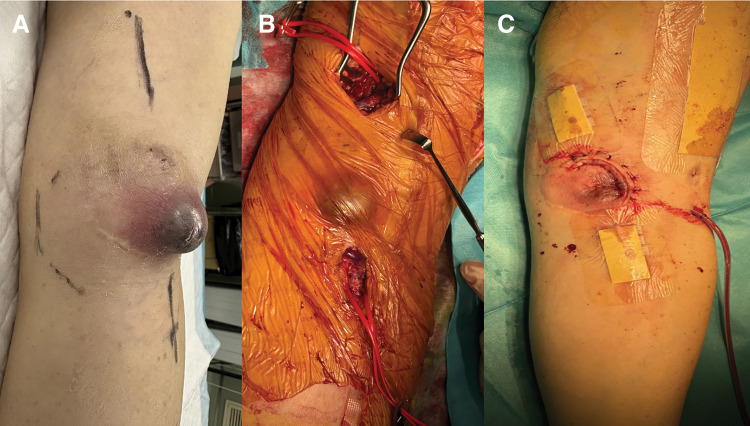
(**A**) Infected SBA aneurysm. (**B**) Bypass using the radial vein. (**C**) Post-resection of the SBA aneurysm. SBA: superficialized brachial artery

A 59-year-old male on dialysis for 13 years with hypertension, hyperlipidemia, and prior stroke presented with a rapidly enlarging SBA aneurysm and purulent discharge (**Fig. 3**). He was diagnosed with an infected pseudoaneurysm. Due to poor great saphenous vein quality, a brachial artery bypass was performed using the radial vein of the upper arm, and the pseudoaneurysm was resected.

## Discussion

### Significance and risks of the superficial brachial artery

The SBA is an effective alternative when autogenous vein creation is not feasible, especially in patients with heart failure or elderly dialysis patients.^1)^ However, repeated punctures over time lead to thinning of the tunica media and intimal hyperplasia, making the artery fragile. External factors such as puncture trauma, poor hemostasis, or infection can cause true or pseudoaneurysms. Early diagnosis and intervention are critical to prevent life-threatening rupture.

In this study, more than half of the cases involved infection, particularly in pseudoaneurysms, suggesting that minor infections at puncture sites can rapidly lead to vessel wall weakening. Anatomically, the superficialized artery lies just beneath the skin, making it more susceptible to direct skin-to-vessel infection compared to deeper shunts.^2)^

### Advantages of revascularization

Compared with simple ligation of the brachial artery, surgical revascularization offers 2 advantages. First, it can prevent ischemic complications of the upper extremity.

Second, it allows the creation of a vascular access in the affected limb. The reported incidence of hand ischemia after brachial artery ligation ranges from 0% to 20%,^3)^ and that of sensory disturbance in the hand is approximately 11%.^4)^ Ligation at a proximal site of the brachial artery has been identified as a high-risk factor for ischemic complications. In the present series, vascular access was created in 4 limbs (50%) that underwent revascularization—using an autologous vein in 1 case, a prosthetic graft in 2 cases, and an SBA in 1 case. For younger patients and for those in whom the contralateral arm cannot be used for access creation, such as individuals with a pacemaker, preserving the potential for vascular access in the affected limb is a significant benefit.

### Material and technique selection for revascularization

Material choice depends heavily on infection status. In infected fields, autologous great saphenous vein is preferred for its biocompatibility and infection resistance.^5)^ In our series, autologous veins were used successfully in infected cases. Prosthetic grafts offer procedural flexibility and are useful in emergencies, but they carry a higher infection risk and require thorough debridement and strict infection control.^6)^

Bovine pericardial patch angioplasty is gaining attention as an option for localized arterial wall defects, preserving flow without a complete bypass and reducing invasiveness.^7)^ However, in our study, patch repair resulted in delayed wound healing and pseudoaneurysm formation, requiring reoperation. Wound complications may be associated with the use of xeno-pericardial patch at infected sites. The outcomes of bovine pericardial patches for infected SBA aneurysms remain unclear.

### Infection control and postoperative management

Dialysis patients are at higher risk for infection due to immunosuppression, malnutrition, and repeated puncture. The superficial location of the SBA further increases the risk of wound infections spreading deeper.

All infected cases in this study were treated with thorough debridement and, when indicated, preoperative antibiotics. Successful revascularization in infected fields requires adequate resection, appropriate graft selection (autologous if possible), and careful postoperative access planning in collaboration with dialysis teams to prevent reinfection and recurrence.^8)^

## Conclusion

Bypass surgery for SBA aneurysms produced favorable outcomes. Ulnar-route bypass using the great saphenous vein is particularly useful for infected aneurysms or lesions extending across the elbow joint.
